# Salt- and pH-Dependent Thermal Stability of Photocomplexes from Extremophilic Bacteriochlorophyll *b*-Containing *Halorhodospira* Species

**DOI:** 10.3390/microorganisms10050959

**Published:** 2022-05-02

**Authors:** Yukihiro Kimura, Kazuna Nakata, Shingo Nojima, Shinji Takenaka, Michael T. Madigan, Zheng-Yu Wang-Otomo

**Affiliations:** 1 Department of Agrobioscience, Graduate School of Agriculture, Kobe University, Nada, Kobe 657-8501, Japan; kn.tea0818@gmail.com (K.N.); nojimashingo@gmail.com (S.N.); stakenaka@people.kobe-u.ac.jp (S.T.); 2 Department of Microbiology, Southern Illinois University, Carbondale, IL 62901, USA; madigan@micro.siu.edu; 3 Faculty of Science, Ibaraki University, Mito 310-8512, Japan

**Keywords:** purple phototrophic bacteria, extremophile, *Halorhodospira halochloris*, *Halorhodospira abdelmalekii*, light-harvesting 1 reaction center, bacteriochlorophyll *b*, thermal stability, salt- and pH-dependence

## Abstract

*Halorhodospira* (*Hlr.*) species are the most halophilic and alkaliphilic of all purple bacteria. *Hlr. halochloris* exhibits the lowest LH1 *Q*_y_ transition energy among phototrophic organisms and is the only known triply extremophilic anoxygenic phototroph, displaying a thermophilic, halophilic, and alkaliphilic phenotype. Recently, we reported that electrostatic charges are responsible for the unusual spectroscopic properties of the *Hlr. halochloris* LH1 complex. In the present work, we examined the effects of salt and pH on the spectroscopic properties and thermal stability of LH1-RCs from *Hlr. halochloris* compared with its mesophilic counterpart, *Hlr. abdelmalekii*. Experiments in which the photocomplexes were subjected to different levels of salt or variable pH revealed that the thermal stability of LH1-RCs from both species was largely retained in the presence of high salt concentrations and/or at alkaline pH but was markedly reduced by lowering the salt concentration and/or pH. Based on the amino acid sequences of LH1 polypeptides and their composition of acidic/basic residues and the Hofmeister series for cation/anion species, we discuss the importance of electrostatic charge in stabilizing the *Hlr. halochloris* LH1-RC complex to allow it to perform photosynthesis in its warm, hypersaline, and alkaline habitat.

## 1. Introduction

Purple phototrophic bacteria have evolved to adapt to various conditions, and some species thrive at extremes of temperature, salinity, and pH [1,2]. *Halorhodospira* (*Hlr*.) *halochloris* (formerly *Ectothiorhodospira halochloris*) is a purple sulfur bacterium (*Gammaproteobacteria*) that was isolated from warm and extremely saline and alkaline Egyptian soda lakes [3]. *Hlr. halochloris* is the only known triply extremophilic anoxygenic phototroph, displaying a thermophilic, halophilic, and alkaliphilic phenotype; the organism grows optimally at 47–50 °C, pH 8–9, and 14–27% (*w*/*v*) salinity [4]. *Hlr. abdelmalekii* (formerly *Ectothiorhodospira abdelmalekii*), isolated from alkaline soda lakes of the Wadi Natrun in Egypt, is a mesophilic counterpart of *Hlr. halochloris*, growing optimally at 30–40 °C, pH 8.5, and 14–16% (*w*/*v*) salinity [5]. Both of these phototrophs assemble their light-harvesting machinery with bacteriochlorophyll (BChl) *b* [2,3,5,6,7,8,9,10,11,12]; because of this, they are part of a distinct minority (< 5%) of known purple bacteria. The light-harvesting 1 (LH1) complexes of BChl *b*-containing purple bacteria exhibit *Q*_y_ absorption bands at wavelengths beyond 1000 nm whose energy is much lower than that absorbed by BChl *a*-containing purple bacteria, whose LH1 complexes typically absorb near 880 nm [13]. 

Among purple phototrophic bacteria, several species are thermophilic. For Bchl *a*-containing species, these include *Thermochromatium* (*Tch*.) *tepidum* from the Mammoth Hot Springs of Yellowstone National Park, USA [14], *Allochromatium* (*Alc*.) *tepidum* from a sulfidic hot spring in New Zealand [15,16], *Caldichromatium* (*Ccm.*) *japonicum* from the Nakabusa hot springs in the Northern Alps of Japan [17], and *Hlr. halophila* from Summer Lake, a seasonally dry hypersaline lake in Oregon (USA) [18]. For BChl *b*-containing species, these include *Blastochloris* (*Blc.*) *tepida,* isolated from a hot spring in New Mexico (USA) [9,11], and *Hlr. halochloris* [3]. Several factors have been linked to the thermal stability of the LH1-RC complexes of these unusual phototrophs. The first of these is Ca^2+^. Thermal stability of the *Tch. tepidum* LH1-RC is achieved by the binding of Ca^2+^ to LH1 polypeptides in a stoichiometric ratio of 1:1 (16 Ca^2+^ to 16 αβ-polypeptides) [19,20], as revealed by a high-resolution X-ray crystallographic structural analysis of the complex [21]. Ca^2+^-dependent thermal stabilization was also reported for the *Alc. tepidum* LH1-RC [22]; however, in this case, the stoichiometric ratio of Ca^2+^ to αβ-polypeptide was lower (0.375) than that of *Tch. tepidum* [22] and has been confirmed to be 6 Ca^2+^ to 16 αβ-polypeptides in the cryo-EM structure of the *Alc. tepidum* LH1-RC [23]. In this species, each LH1 ring contains multiple pairs of α- and β-polypeptides, but only six α1-polypeptides bind Ca^2+^ with β1- or β3-polypeptides to form the Ca^2+^-binding sites that reinforce the complex. As for the other thermophiles that contain Bchl *a* (*Hlr. halophila* and *Ccm. japonicum*), the factors responsible for LH1-RC thermal stability remain to be determined.

An alternative factor conferring LH1-RC thermal stability is the presence of carotenoids with extended conjugation. The purple nonsulfur bacterium *Blc. tepida* grows optimally at 42 °C and up to 47 °C [9,11]. However, in contrast to *Tch. tepidum*, Ca^2+^ is neither present in, nor has any effect on, the thermostability of *Blc. tepida* LH1-RC [24]. In comparative studies of *Blc. tepida* with its mesophilic counterpart, *Blc. viridis*, it was discovered that lycopene family carotenoids with elongated conjugations are produced by the thermophile and that these strengthen hydrophobic interactions with LH1-RC proteins; as a result, the *Blc. tepida* complexes remain functional at higher temperatures than is possible in *Blc. viridis*.

In a previous study, we reported salt- and pH-sensitive interconvertible LH1 *Q*_y_ band shifts of the purified LH1-RC complex from *Hlr. halochloris* [25] and demonstrated that the native spectral properties of the LH1-RC were maintained in the presence of high concentrations of salts and/or at alkaline pH (>7). During these experiments, it was observed that lowering the salt concentration and/or the pH induced *Q*_y_ blue-shifts and decreased the band intensities, suggesting that the thermal stability of the *Hlr. halochloris* LH1-RC complex might also be controlled by salt or pH. Therefore, in the present study, we systematically examined the effects of salts and pH on the thermal stability of the LH1-RC complex from *Hlr. halochloris* and compared them with those from its mesophilic counterpart, *Hlr. abdelmalekii*. Based on our experimental results, along with comparative sequence analyses of relevant LH1-RC proteins, the molecular strategies employed by *Hlr. halochloris* to maintain functional LH1-RC complexes are more fully revealed and are discussed in relation to strategies employed by other purple bacteria.

## 2. Materials and Methods

### 2.1. Growth Conditions and Preparation of LH1-RC Complexes

*Hlr. halochloris* strain DSM 1059 and *Hlr. abdelmalekii* strain DSM 2110 were grown photosynthetically (anoxic/light) at 43 °C and 28 °C, respectively, under illumination of a 60 W incandescent lamp for 7–10 days in the medium of Imhoff and Trüper [3] with slight modifications [25]. The harvested cells suspended in 20 mM Tris-HCl buffer (pH 8.5) were disrupted by sonication (Sonopuls HD3200, Bandelin, Berlin, Germany) and ultracentrifuged at 195,000× *g* for 60 min (L60, Beckman) to obtain photosynthetic membranes (chromatophores). The latter were solubilized with 1.0% (*w*/*v*) *n*-dodecyl β-d-maltopyranoside (DDM) at ambient temperature and ultracentrifuged at 195,000× *g* for 60 min to extract crude LH1-RCs. The supernatant was loaded onto an anion exchange column (Toyopearl DEAE-650s, TOSOH) equilibrated with 20 mM Tris-HCl (pH 8.5) and 0.08% (*w*/*v*) DDM at 4 °C. LH1-RC complexes were eluted with a linear gradient of NaCl concentration (100–400 mM) and the fractions with A_LH1 Qy_/A_280_ ratios > 0.8 were collected. Salt concentrations were adjusted by extensive washing of the purified *Hlr. halochloris* and *Hlr. abdelmalekii* LH1-RCs with a buffer containing 20 mM Tris-HCl (pH 8), 0.08% DDM and an indicated concentration of salts (0–1 M) by ultrafiltration (Amicon Ultra-15, Millipore). 

### 2.2. Evaluation of Thermal Stability

Absorption spectra were recorded on a UV-mini 1200 (Shimadzu) and V-730Bio spectrophotometer (JASCO). Thermal degradation of LH1-RC complexes was monitored as reductions in LH1 *Q*_y_ band intensities after incubation at 45 °C for 0–60 min. For all experiments, sample concentrations were normalized with respect to the *Q*_x_ band at 600 nm because this band was shown to be unaffected by salt concentration or pH.

## 3. Results

### 3.1. NaCl and pH Effects on Spectroscopic Properties of LH1-RC Complexes from Halorhodospira Species

**Figure 1:**
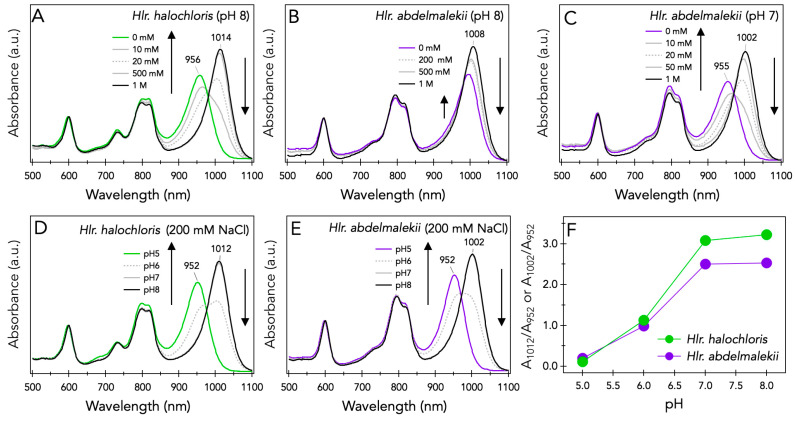
Absorption spectra of the LH1-RC complexes from *Hlr. halochloris* (**A**,**D**) and *Hlr. abdelmalekii* (**B**,**C**,**E**) at pH 8.5 in the presence of indicated concentration of NaCl at pH 8 (**A**,**B**) or pH 7 (**C**), and those at different pHs in the presence of 200 mM of NaCl (**D**,**E**). Panel (**F**) shows plots of the ratio of two LH1 *Q*_y_ band intensities (A_1012_/A_952_ for *Hlr. halochloris* and A_1002_/A_952_ for *Hlr. abdelmalekii*) at various pHs.

Figure 1A,B show the absorption spectra of desalted LH1-RCs from *Hlr. halochloris* and *Hlr. abdelmalekii* at pH 8 after supplementation with 0–1 M NaCl. For *Hlr. halochloris* (Figure 1A), the desalted LH1 *Q*_y_ band appeared at 956 nm in the absence of NaCl and was red-shifted as the NaCl concentration increased, reaching 1014 nm at 1 M NaCl. The salt-dependent and interconvertible spectral changes of the *Hlr. halochloris* LH1-RCs were largely comparable to those reported previously [25] but were significantly different from those observed for the mesophilic *Hlr. abdelmalekii* LH1-RCs, as shown in Figure 1B. At pH 8, the desalted LH1 *Q*_y_ band of this species was not completely blue-shifted to ~955 nm. However, a salt-dependent and interconvertible spectral change was also confirmed for *Hlr. abdelmalekii* LH1-RCs when the buffer pH was decreased to 7 (Figure 1C). The LH1 Q_y_ band appeared at 955 nm upon desalting and was red-shifted as the NaCl concentration increased, reaching 1002 nm at 1 M NaCl. These results suggest that although there may be a common mechanism responsible for the salt-dependent LH1 spectral shifts of *Hlr. halochloris* and *Hlr. abdelmalekii*, the salt requirements for maintaining the native spectral properties of the LH1-RCs differ in the two *Halorhodospira* species at a given pH. 

Figure 1D,E exhibit the absorption spectra of the purified LH1-RCs from *Hlr. halochloris* and *Hlr. abdelmalekii*, respectively, at pH 5–8 in the presence of 200 mM NaCl. At pH 8, the LH1 *Q*_y_ band appeared at 1012 nm for *Hlr. halochloris* and 1002 nm for *Hlr. abdelmalekii*, and both *Q*_y_ bands were blue-shifted to 952 nm at lower pH. The pH-dependent spectral changes were interconvertible, not only for *Hlr. halochloris* (Figure 1D) [25,26] but also for *Hlr. abdelmalekii* (Figure 1E). In addition, their pH requirements were nearly identical, as can be seen in plots of the ratio of two LH1 *Q*_y_ band intensities (A_1012_/A_952_ for *Hlr. halochloris* and A_1002_/A_952_ for *Hlr. abdelmalekii*) at various pHs (Figure 1F). The salt- and pH-dependent spectral changes of these *Halorhodospira* species were nonspecific to coexisting ion species and their valences [25], and distinct from the Ca^2+^-specific spectral changes of several BChl a-containing purple sulfur bacteria, including *Tch. tepidum* [27], *Alc. tepidum* [22], and *Thiorhodovibrio* strain 970 [28]. These results also support our previous conclusion that electrostatic charges control the LH1 *Q*_y_ transition energies of LH1-RC complexes from *Hlr. halochloris* [25], as they likely do in *Hlr. abdelmalekii* as well.

### 3.2. NaCl and pH Effects on the Thermal Stability of LH1-RC Complexes from Halorhodospira Species 

**Figure 2:**
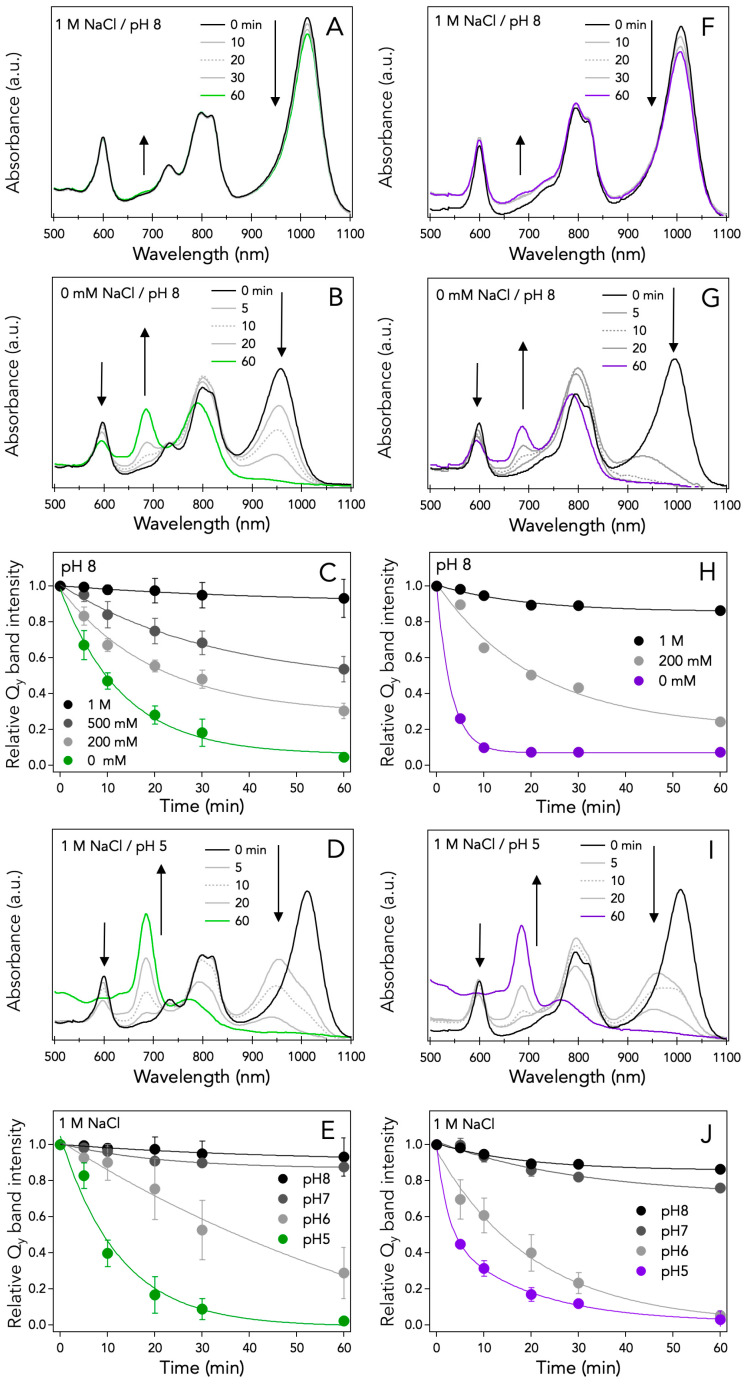
Absorption spectra of the LH1-RCs from *Hlr. halochloris* (**A**,**B**) and *Hlr. abdelmalekii* (**F**,**G**) at pH 8 in the presence of 1 M NaCl (**A**,**F**) or in the absence of NaCl (**B**,**G**) after incubation at 45 °C for indicated time. The time profiles of the relative *Q*_y_ band intensities for the *Hlr. halochloris* (**C**) and *Hlr. abdelmalekii* (**H**) LH1-RCs, respectively, incubated at 45 °C and pH 8 in the presence of indicated concentration of NaCl. Absorption spectra of the LH1-RCs from *Hlr. halochloris* (**D**) and *Hlr. abdelmalekii* (**I**) at pH 5 in the presence of 1 M NaCl. The relative *Q*_y_ band intensities of the *Hlr. halochloris* (**E**) and *Hlr. abdelmalekii* (**J**) LH1-RCs after incubation at 45 °C for 60 min at indicated pH in the presence of 1 M NaCl.

To better understand the relation between electrostatic charge and protein stability, the effects of salt and pH on the thermal stability of LH1-RCs from *Hlr. halochloris* and *Hlr. abdelmalekii* were examined. Figure 2A shows the rate of thermal degradation of the *Hlr. halochloris* LH1-RC upon incubation at 45 °C and pH 8 for 0–60 min in the presence of 1 M NaCl. Over time, the LH1 *Q*_y_ band intensity at 1014 nm decreased slightly and a faint band increased at 680 nm, assignable to oxidized BChl *b* decomposed from the LH1 complexes [26]. However, in the absence of NaCl, the LH1 *Q*_y_ band (blue-shifted to 952 nm, Figure 1B) steadily decreased to near zero after 1 h while concomitantly, the oxidized BChl b band at 680 nm steadily increased (Figure 2B). Similar experiments were performed at pH 8 with different NaCl concentrations, and their relative *Q*_y_ band intensities were plotted against incubation time in Figure 2C. As can be seen, the thermal stability of *Hlr. halochloris* LH1-RCs was largely retained in the presence of 1 M NaCl but markedly reduced as NaCl concentrations were decreased, indicating that the photocomplexes of this species require salt for thermal stability. 

In additional experiments, the effects of pH on the thermal stability of *Hlr. halochloris* LH1-RCs suspended in 1 M NaCl were examined. Thermal stability was largely retained at pH 8 (Figure 2A) but was much reduced at pH 5 (Figure 2D); in the latter, the LH1 *Q*_y_ band was virtually eliminated and the oxidized BChl *b* band at 680 nm reached its maximum. Moreover, thermal decomposition of *Hlr. halochloris* LH1-RCs at acidic pH (Figure 2D) was more pronounced than that at reduced NaCl concentrations (Figure 2B), as deduced from the 680 nm band intensity of oxidized BChl *b*. Similar experiments were performed in the presence of 1 M NaCl at different pH values, and relative *Q*_y_ band intensities are plotted against incubation time in Figure 2E. The data show that thermal stability is largely retained at pH 7 but markedly reduced as the pH drops below neutrality, demonstrating a pH-dependent thermal stability of *Hlr. halochloris* LH1-RCs. Thus, as one might expect considering the geochemical conditions in the habitat of *Hlr. halochloris* [3], the thermal stability of this organism’s photocomplexes is greatest at both elevated pH and salt concentration.

These salt- and pH-dependent effects on the thermal stabilization of purified *Hlr. halochloris* LH1-RCs were confirmed using photosynthetic membranes (chromatophores) in which LH1-RCs remain embedded in the lipid bilayer (Figure S1). The relative *Q*_y_ band intensities of *Hlr. halochloris* chromatophores were evaluated after incubation at the indicated pH and temperature for 60 min in the presence (Figure S1A) or absence (Figure S1B) of 4 M NaCl. The relative *Q*_y_ band intensities were almost completely diminished in the absence of NaCl (Figure S1B) or at low pH in the presence of 4 M NaCl (Figure S1A). These results suggest that the salt- and pH-dependent thermal stability of photocomplexes in this species is not closely associated with the transmembrane regions, but instead, with the surfaces of the LH1s and/or RCs where solvent molecules are readily accessible. 

Similar studies of LH1-RC thermal degradation were performed on the mesophilic *Hlr. abdelmalekii* to compare it to that of *Hlr. halochloris*. Figure 2F shows the thermal degradation of the *Hlr. abdelmalekii* LH1-RC upon incubation at 45 °C and pH 8 in the presence of 1 M NaCl. Similar to *Hlr. halochloris*, the LH1 *Q*_y_ band intensity at 1008 nm was slightly decreased along with a faint increase of the oxidized BChl *b* band at 680 nm. By contrast to *Hlr. halochloris*, thermal decomposition of *Hlr. abdelmalekii* LH1-RCs was rapid in the absence of NaCl at pH 8 (Figure 2G); even with salt present at pH 5, degradation was rapid, as in *Hlr. halochloris* (Figure 2D,I). The degrees of thermal stability of *Hlr. halochloris* and *Hlr. abdelmalekii* LH1-RCs seemed comparable at pH 8 if salt was present (Figure 2A,F). However, significant differences in thermal stability were apparent as salt levels decreased at pH 8 (Figure 2H) or when pH decreased in the presence of salt (Figure 2J); these differences are clearly seen in the life times obtained with a first-order kinetics analysis of each decay (Tables S1 and S2). These results indicate an overall greater thermal stability for *Hlr. halochloris* LH1-RCs compared with *Hlr. abdelmalekii* LH1-RCs. These data therefore indicate that not only are the spectral properties of LH1 complexes in both *Halorhodospira* species controlled by electrostatic charges, but also their thermal stability.

### 3.3. Effect of Other Salts on Thermal Stability of LH1-RCs from Hlr. halochloris

**Figure 3:**
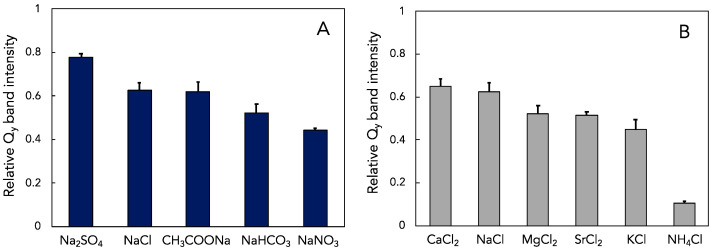
The relative *Q*_y_ band intensities of the *Hlr. halochloris* LH1-RCs in the presence of 500 mM of various sodium salts (**A**) or chlorides (**B**) after incubation at 45 °C and pH 8 for 60 min.

To further probe how electrostatic charge influences the thermal stability of *Hlr. halochloris* LH1-RC complexes, we tested the capacity of different sodium (Figure 3A) or chloride (Figure 3B) salts to substitute for NaCl. Upon incubation at 45 °C for 60 min in the presence of 500 mM of each sodium salt (Figure 3A), the LH1 *Q*_y_ band intensity of *Hlr. halochloris* was largely retained by Na_2_SO_4_, only moderately affected by NaCl and CH_3_COONa, and was reduced by approximately half with NaHCO_3_ and NaNO_3_. Since sodium ions are present in each of these salts, the effect of each on thermal stability is attributed to anions in the order of SO_4_^2−^ > Cl^−^ ≈ CH_3_COO^−^ > HCO_3_^−^ > NO_3_^−^. This order is in general agreement with the Hofmeister series—a classification of ions in order of their ability to solubilize proteins or stabilize secondary and tertiary structures of proteins [29,30]. A standard Hofmeister anion series is SO_4_^2−^ > HPO_4_^2−^ > CH_3_COO^−^ > Cl^−^ > NO_3_^−^ > ClO_3_^−^ > I^−^ > ClO_4_^−^ > SCN^−^ [31], in which the most potent stabilizing anions are located on the left side of each pair. The Hofmeister series also orders a standard cation series as NH_4_^+^ > K^+^ > Na^+^ > Li^+^ > Mg^2+^ > Ca^2+^ [31], in which the most effective stabilizing cations are also positioned on the left side of each pair. Curiously, however, the Hofmeister cation series is in opposition to the results obtained with different chloride salts; the ability of cations to stabilize the *Hlr. halochloris* LH1-RCs was in the order of Ca^2+^ > Na^+^ > Mg^2+^ > Sr^2+^ > K^+^ >> NH_4_^+^ (Figure 3B). 

## 4. Discussion

### 4.1. Effects of Salt Concentration and pH on the Spectral Properties of the LH1-RC Complexes from Halorhodospira Species

**Figure 4:**
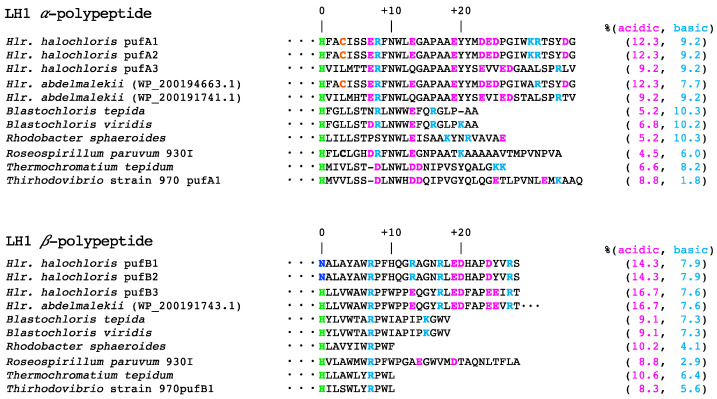
Amino acid sequences of the LH1 α- and β-polypeptides from several purple phototrophic bacteria. The sequences are aligned relative to the His residues (His^0^, green) coordinating BChl molecules. Acidic (Asp, Glu) and basic (Arg, Lys) residues are highlighted with magenta and cyan, respectively. Unique Cys residues in the vicinity of His^0^ are colored with orange. The numbers of right side represent percentages of acidic and basic residues in each polypeptide.

In a previous study, we proposed that two factors may responsible for the salt- and pH-dependent spectral changes of the *Hlr. halochloris* LH1-RCs [25]. The first is the axial ligand for Mg^2+^ of BChl *b* bound to LH1 β-polypeptide. Figure 4 compares the amino acid sequences of the LH1 α- or β-polypeptides from several purple bacteria including *Hlr. halochloris* and *Hlr. abdelmalekii.* The conserved His^0^ residues in LH1 α-polypeptides are a plausible binding site for α-BChl molecules, and this is also true for most LH1 β-polypeptides other than PufB1 and PufB2 of *Hlr. halochloris*, in which Asn is substituted for the β-BChl binding His^0^ [32,33,34]. Considering that PufB1/PufB2 and PufB3 were comparably expressed, it was hypothesized that the amide oxygen of the Asn side chain modulates its LH1 *Q*_y_ transition energy by serving as an axial ligand for the *Hlr. halochloris* LH1 β-BChl *b* [25]. However, this hypothesis does not hold for *Hlr. abdelmalekii*, because such Asn residues are absent from its LH1 β-polypeptide (NCBI Reference Sequence: WP_200191743.1), a polypeptide almost identical to PufB3 of *Hlr. halochloris* (Figure 4). 

A second factor that may affect salt- and pH-dependent spectral changes in *Hlr. halochloris* LH1-RCs is LH1 α-Cys^+3^ [25]. Such a residue is rarely present near an α-BChl *b*-binding His^0^ and is replaced with Leu or Val in most purple bacteria other than *Roseospirillum parvum* [33,34]. Based on the pKa values of the Cys side chain (ranging from 2.9 to 9.8 inside proteins) [35] and its close association with LH1 BChl molecules [36], we proposed the side chain thiol/thiolate of α-Cys^+3^ as a candidate for modulating the point-charge around molecules of BChl *b* that trigger the pH-dependent interconvertible LH1 *Q*_y_ band shifts of *Hlr. halochloris* [25]. The present study demonstrated that the *Hlr. abdelmalekii* LH1-RCs exhibited salt- and pH-dependent interconvertible spectral changes as well (Figure 1). In this respect, one of the LH1 α-polypeptides from *Hlr. abdelmalekii* (NCBI Reference Sequence: WP_200194663.1) is almost identical to PufA1/A2 of *Hlr. halochloris* and has the α-Cys^+3^ residue (Figure 4), although the expression of this (or any *Hlr. abdelmalekii* LH1 polypeptides) is unknown [37]. However, considering these results, we suggest that the α-Cys^+3^ thiolate anions present at alkaline pH contribute to the ultra-red-shift of the LH1 *Q*_y_ band while the protonated form at acidic pH results in a LH1 *Q*_y_ band blue-shift. Under high salt conditions, the thiolate anions may be surrounded by cations, thus disturbing the protonation of the thiolate anions and maintaining the red-shifted LH1 *Q*_y_ bands. By contrast, protonation of α-Cys^+3^ thiolate anions would be favored when cations were limiting, leading to blue-shifts of the LH1 *Q*_y_ band.

### 4.2. Effects of Amino Acid Compositions on the Thermal Stability of the LH1-RC Complexes from Halorhodospira Species

It is known that halophilic *Archaea* such as *Halobacterium* store high intracellular concentrations of inorganic salts (primarily K^+^) for osmoregulation purposes under hypersaline conditions by balancing the excess positive charges present from molar concentrations of K^+^ [38]. In contrast, most halophilic *Bacteria* and eukaryotes accumulate organic compatible solutes [39] instead of inorganic salts for osmoregulation. As for *Hlr. halochloris*, it accumulates glycine betaine, with minor amounts of ectoine and trehalose as compatible solutes [40,41,42] but does not accumulate KCl [43]. Furthermore, phototrophic growth of *Hlr. halochloris* cells was almost completely suppressed when NaCl (>3 M) in the medium was depleted or replaced with KCl (Figure S2). These findings suggest that a high concentration of NaCl is indispensable for physiological regulation in *Hlr. halochloris* cells and that NaCl is not functionally replaceable with KCl. 

The salt- and pH-dependent stabilization of the LH1-RC from *Halorhodospira species* was confirmed not only in purified LH1-RCs but also in photosynthetic membranes (Figure S1). This indicates that surfaces of the LH1-RCs in contact with solvents (rather than transmembrane domains) are the regions of the proteins that sense and respond to changes in salt concentration and pH. In addition, LH1 BChl molecules that exhibit a salt- and pH-dependent spectral change are located on the C-terminal side (periplasmic side) of the photosynthetic membranes. Therefore, we focused our analyses on the amino acid composition of LH1 polypeptides (Figure 4) and the RC C-subunits (Figure S3) from various purple bacteria. It has been observed that halophilic proteins contain increased levels of negative charges due to the presence of acidic amino acids on their surfaces [38,44,45,46,47]. In agreement with this, the contents of acidic residues in both *Hlr. halochloris* and *Hlr. abdelmalekii* LH1 α- and β-polypeptides are significantly greater than those in non-halophilic purple bacteria, and the excess acidic residues are predominantly present in their C-terminal regions (Figure 4). In addition to LH1 polypeptides, the C-subunits of *Halorhodospira* RCs also exhibit high contents of acidic residues compared with those of nonhalophilic purple bacteria (Figure S3). Collectively, these acidic residues present in the LH1-RC surfaces exposed to solvents may be necessary for the *Hlr. halochloris* and *Hlr. abdelmalekii* LH1-RC complexes to function at the high salinities experienced in their habitats. 

The contents of acidic residues in the LH1-RC complexes of both halophiles are also much greater than those of basic residues (Figure 4 and Figure S3). These views are compatible with the observation that halophilic proteins show a biased amino acid composition on the protein surface, with a large excess of acidic residues and a reduced number of basic residues compared to corresponding proteins from nonhalophiles [46,48]. The highly negative charges of the abundant acidic residues on the halophilic protein surfaces improve their solubility and flexibility at high salt concentrations and allow them to function normally. By contrast, their electrostatic repulsions are thought to be a major factor responsible for the pronounced instability of proteins at lower salt concentrations [45], consistent with the present salt-dependent destabilization of the *Hlr. halochloris* and *Hlr. abdelmalekii* LH1-RC complexes (Figure 2C,H). Therefore, cations of salts may help stabilize the excess negative charges on the surfaces of halophilic LH1-RCs. 

However, it is also known that highly negative charges in halophilic proteins can be compensated with tightly bound water dipoles, rather than excess cations, by forming water shells and hydrogen bonds to help maintain protein structure [38,49,50]. Water shells may therefore protect proteins from attack by exogenous molecules at higher temperature and contribute to their thermal stability. Thus, it is also possible that water shells exist in the *Hlr. halochloris* and *Hlr. abdelmalekii* LH1-RCs. If true, the much-reduced thermal stability of the halophilic LH1-RCs observed at acidic pH could be due to the loss of water shells upon protonation of surface acidic residues. Furthermore, the significant difference observed in the thermal stability of the thermophilic and mesophilic LH1-RCs (Figure 2) could be due to the slightly higher ratio of acidic residues in the RC C-subunit and/or LH1 polypeptides of *Hlr. halochloris* compared with those of *Hlr. abdelmalekii*. Such water shells were confirmed in a high-resolution X-ray crystallographic LH1-RC structural analysis of *Tch. tepidum*—a thermophile, as opposed to a halophile—in which a number of water molecules cover the periplasmic and cytoplasmic surfaces of the LH1-RC complex as water shells [21]. To prove this hypothesis, high-resolution 3D structures of the *Hlr. halochloris* and *Hlr. abdelmalekii* LH1-RCs would be needed.

### 4.3. Effects of Anions and Cations on the Thermal Stability of the LH1-RC Complexes from Halorhodospira Species

Halophilic proteins maintain functionally active conformations in the presence of high concentrations of kosmotropic ions (small size, high surface charge density, strong hydration and forming water structures) but become inactive in the presence of high concentrations of chaotropic salts (large size, low surface charge density, weak hydration and breaking water structures) [30,51]. These high concentrations of coexisting anions and cations may affect the water structure on halophilic proteins, as discussed in Section 4.2. In the present study, the stabilizing effect of anions was in the order of SO_4_^2−^ > Cl^−^ ≈ CH_3_COO^−^ > HCO_3_^−^ > NO_3_^−^ (Figure 3), consistent with the Hofmeister series [29,30,31]. It can thus be concluded that kosmotropic anions function as stabilizing factors in the *Hlr. halochloris* LH1-RCs. By contrast, the Hofmeister cation series was in opposition to our results obtained with different chloride salts (Figure 3). Given that anions appear to have a larger effect on protein stability than do cations [30], the thermal stability of the *Hlr. halochloris* LH1-RCs is generally consistent with that predicted from the Hofmeister anion series. However, our results with different cations could signal that yet another mechanism contributes to the stability of halophilic proteins. Regarding the Hofmeister series, it was proposed that specific interactions between ions and proteins and ions and water molecules directly contacting the proteins may be as important as the changes that occur in the general water structure [30,31,51]. Therefore, the specific interactions of cations and the LH1-RC and/or possible water shells may be crucial for understanding the stability of LH1-RCs in both *Halorhodospira* species. For example, a possible explanation for the enhanced photocomplex stability observed with Ca^2+^ (Figure 3B) is that a part of acidic residues largely present in the C-terminal side of the *Hlr. halochloris* LH1-RCs may form specific binding sites for Ca^2+^ that reinforce protein structure in a manner similar to that of the Ca^2+^-binding LH1-RC proteins present in several purple sulfur bacterial complexes [21,23,52]. To confirm this hypothesis, highly resolved structural information is required for the LH1-RC complexes from these *Halorhodospira* species.

## Data Availability

Not applicable.

## Supplementary Materials

The following supporting information can be downloaded at: https://www.mdpi.com/article/10.3390/microorganisms10050959/s1, Table S1: Lifetimes for the thermal degradation of the LH1-RC complexes from *Hlr. halochloris* and *Hlr. abdelmalekii* at pH 8 in the presence of indicated concentration of NaCl; Table S2: Lifetimes for the thermal degradation of the LH1-RC complexes from *Hlr. halochloris* and *Hlr. abdelmalekii* in the presence of 1M NaCl at various pHs; Figure S1: The relative *Q*_y_ band intensities of *Hlr. halochloris* chromatophores in the presence of 4 M NaCl (A) or in the absence of NaCl (B) after incubation at the indicated pH and temperature for 60 min; Figure S2: Phototrophic growth curves for *Hlr. halochloris* in the presence of 3 M NaCl (black) or 3M KCl (purple) or in the absence of salts (gray) monitored as LH1 *Q*_y_ peak intensity at 1020 nm. Photos of *Hlr. halochloris* cultures grown under the indicated condition for 10 days are shown in the inset; Figure S3. Amino acid sequences of the RC C-subunits from several extremophilic purple phototrophic bacteria. Acidic (Asp, Glu) and basic (Arg, Lys) residues are highlighted with magenta and cyan, respectively. The numbers on the right side represent percentages of acidic and basic residues in each subunit.
