# Supplementary material for: Salt- and pH-Dependent Thermal Stability of Photocomplexes from Extremophilic Bacteriochlorophyll b-Containing Halorhodospira Species
Source: Microorganisms. 2022 May 2;10(5):959. doi: 10.3390/microorganisms10050959 (PMC9146400; doi:10.3390/microorganisms10050959)
Supplement: Supplementary file 1 [file microorganisms-10-00959-s001.zip › microorganisms-1678079-supplementary.pdf]

microorganisms

MDPI

Supplementary Materials

Table S1. Lifetimes for the thermal degradation of the LH1-RC complexes from *Hlr. halochloris* and *Hlr. abdelmalekii* at pH 8 in the presence of indicated concentration of NaCl.

	Lifetimes (min)			
	**0 mM NaCl**	**200 mM NaCl**	**500 mM NaCl**	**1 M NaCl**
Hlr. halochloris	15.4	44.2	90.4	825
Hlr. abdelmalekii	3.91	35.2	–	401

Table S2. Lifetimes for the thermal degradation of the LH1-RC complexes from *Hlr. halochloris* and *Hlr. abdelmalekii* in the presence of 1M NaCl at various pHs.

	Lifetimes (min)			
	**pH 5**	**pH 6**	**pH 7**	**pH 8**
Hlr. halochloris	12.2	50.0	439	825
Hlr. abdelmalekii	8.71	21.1	197	401

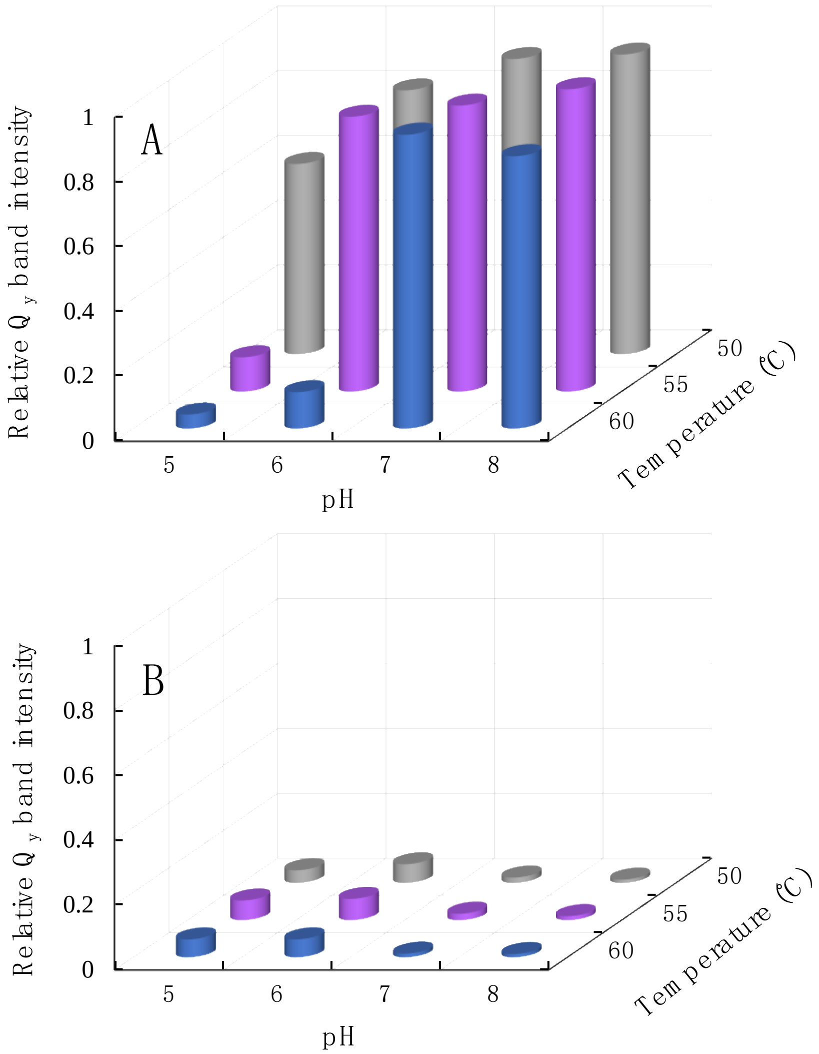

Figure S1. The relative Q_y band intensities of *Hlr. halochloris* chromatophores in the presence of 4 M NaCl (A) or in the absence of NaCl (B) after incubation at the indicated pHs and temperatures for 60 min. Kimura et al.

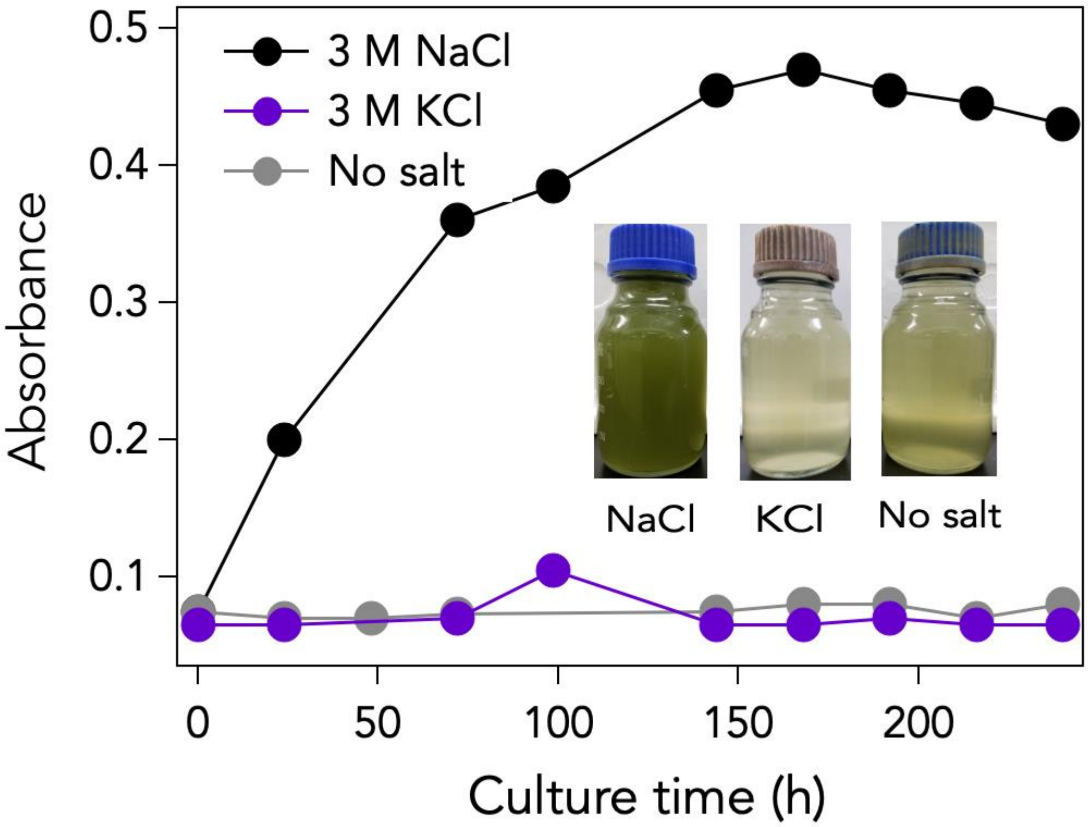

Figure S2. Phototrophic growth curves for *Hlr. halochloris* in the presence of 3 M NaCl (black) or 3M KCl (purple) or in the absence of salts (gray) monitored as LH1 Q_y peak intensity at 1020 nm. Photos of *Hlr. halochloris* cultures grown under the indicated condition for 10 days are shown in the inset. Kimura et al.

%(acidic, basic)

Hlr. halochloris (BAU58341.1)

```
MDGRYLTRSLAVGALAAGFLVVAGCERPPFEQEQTGPRGTGMYVLDNPRILESRLDLHTAPEARPMASED
GERAGDVHENVQVLADLSDEQFWRIKEEMTDWVAGDEGCTYCHTDDLASDEKYQYRVSRDMIEMTRYLNA
NWADTHLTHSNEAGVTCYTCHRGEPIPPASWHSEEESGETRFMTGMGDLQLQNKISSKTAYTAFPRDALD
TFLVGHEGELAIVGEGEGGLRTATTEGVSLREAYEAVGLMMHLSYSLDAGCTLCHNVSRWASWEDSPKER
ETAWHGIRMARDINVNWINPLIDEYPEDADVLGPTGDVGKVSCQTCHNKERRPLYGEEFLELYPELVGEP
DPDFDYLQFGDLGTDLLKGVND
```

(18.3, 8.3)

Hlr. abdelmalekii (NCBI Reference Sequence: WP_200195997.1)

```
MMEGRYLTRSLAVGALAAGFLVVAGCERPPHDAEQTGPRGTGMYVLDNPRILESRLDLHTAPEARPLASE
DGPRAGEVFENVQALADISEAQFWRIKEEMTDWVAPDEGCTYCHTDDLASDEKYQYRVAKDMIKMNRYLN
ANWSETHLASTNQAGVTCYTCHRGEPIPPASWHSESESHLKTGFGDMQLQNLISEKTAYTALPRDALDAF
LVDHQTIPIQGSTGLRTATTPRGKSLHESERSVSLMMHISYALDTGCTLCHNVSRWASWEDSPIGREIAW
HGIRMARDINVNWMIPLTDEYPEDALGPAGDVGKVSCQTCHNKERRPLYGEEFLELYPELVGDPDPDFDY
LQFGDLGTDLLKGSR
```

(15.3, 9.0)

Blc. tepida (NCBI Reference Sequence: WP_126396869.1)

```
MKHMIAKSVATVALASLVSGCFEPPPAISTQTGFRGLSMGEVLHPATVAAKKERDAQYPPALPAVKAEGQ
PVSKVYKNVKVLGDLTEPEFLRTMTAMTEWVSPKEGCTYCHDEADLSSEAKYPFKVARRMLEMTRHINTD
WTSHVAQTGVTCYTCHRGRPVPPYIRYLEPRLPLDNAIKPTFVEADNSGHVVRLAKNTAYSALNYDPFAM
FLANDKREIRFVPQTALPPVGVSRGMERRPLSDAYATFALMMFISDAIGTNCTFCHNPQTFESWGNKSTP
QRAIAWQGIKMTRDLNMNFLSPLKPVYPANRLGAQGEAPMADCRTCHQGVTKPLFGASRMKDYPELGPVK
AAAK
```

(9.0, 12.1)

Tch. tepidum (PDB 5Y5S)

```
MSPAQQLTLPAVIVVASVMLLGCEGPPPGTEQIGYRGVGMENYYNKRQRALSIQANQPVESLPAADSTGP
KASEVYQNVQVLKDLSVGEFTRTMVAVTTWVSPKEGCNYCHVPGNWASDDIYTKVVSRRMFELVRAANSD
WKAHVAETGVTCYTCHRGNPVPKYAWVTDPGPKYPSGLKPTGQNYGSKTVAYASLPFDPLTPFLDQANEI
RITGNAALAGSNPASLKQAEWTFGLMMNISDSLGVGCTFCHNTRAFNDWTQSTPKRTTAWYAIRHVRDIN
QNYIWPLNDVLPASRKGPYGDPLRVSCMTCHQAVNKPLYGAQMAKDYPGLYKTAVTQEALAGSAPASEAA
PAAATEAAPEAPAQEVPAAEAVPAAAEPGAAEAAGSVEPAPVEEVAPAPAAQRL
```

(8.9, 7.7)

Blc. viridis (PDB 6ET5)

```
CFEPPPATTTQTGFRGLSMGEVLHPATVKAKKERDAQYPPALAAVKAEGPPVSQVYKNVKVLGNLTEAEF
LRTMTAITEWVSPQEGCTYCHDENNLASEAKYPYVVARRMLEMTRAINTNWTQHVAQTGVTCYTCHRGTP
LPPYVRYLEPTLPLNNRETPTHVERVETRSGYVVRLAKYTAYSALNYDPFTMFLANDKRQVRVVPQTALP
LVGVSRGKERRPLSDAYATFALMMSISDSLGTNCTFCHNAQTFESWGKKSTPQRAIAWWGIRMVRDLNMN
YLAPLNASLPASRLGRQGEAPQADCRTCHQGVTKPLFGASRLKDYPELGPIKA
```

(8.4, 11.7)

Figure S3. Amino acid sequences of the RC C-subunits from several extremophilic purple phototrophic bacteria. Acidic (Asp, Glu) and basic (Arg, Lys) residues are highlighted with magenta and cyan, respectively. The numbers on the right side represent percentages of acidic and basic residues in each subunit. Kimura et al.
